# Prognostic Value of Novel CARWL Score in Stage IIIC Non-Small-Cell Lung Cancer Patients Undergoing Concurrent Chemoradiotherapy

**DOI:** 10.1155/2024/2803044

**Published:** 2024-06-28

**Authors:** Erkan Topkan, Ahmet Kucuk, Duriye Ozturk, Emine Elif Ozkan, Ali Ayberk Besen, Berrin Pehlivan, Ugur Selek

**Affiliations:** ^1^ Department of Radiation Oncology Baskent University Medical Faculty, Adana, Türkiye; ^2^ Clinic of Radiation Oncology Mersin Education and Research Hospital, Mersin, Türkiye; ^3^ Department of Radiation Oncology Faculty of Medicine Afyonkarahisar Health Sciences University, Afyonkarahisar, Türkiye; ^4^ Department of Radiation Oncology Suleyman Demirel University, Isparta, Türkiye; ^5^ Department of Medical Oncology Medical Park Adana Hospital Medical Faculty, Adana, Türkiye; ^6^ Department of Radiation Oncology Bahcesehir University, Istanbul, Türkiye; ^7^ Department of Radiation Oncology School of Medicine Koc University, Istanbul, Türkiye

## Abstract

**Objectives:**

We explored the prognostic utility of the unique combination of C-reactive-protein-to-albumin ratio (CAR) and significant weight loss (WL > 5%) over the preceding 6 months, namely, the CARWL score, in stage IIIC non-small-cell lung cancer (NSCLC) patients who underwent concurrent chemoradiotherapy (CCRT).

**Methods:**

For each patient, the CAR was calculated using C-reactive protein and albumin measurements obtained on the first day of CCRT: CAR = C-reactive protein ÷ albumin. The availability of an ideal CAR cutoff that may categorize patients into two distinct progression-free (PFS) and overall survival (OS) outcomes was explored by employing receiver operating characteristic (ROC) curve analysis. Patients were additionally divided into two groups based on their status of significant WL according to the well-recognized Delphi criteria. Then, the CARWL score was created by combining all feasible combinations of the CAR and significant WL groupings. The potential links between pretreatment CARWL groups and the post-CCRT OS and PFS outcomes were determined as the primary and secondary endpoints.

**Results:**

This retrospective cohort study comprised a total of 651 stage IIIC NSCLC patients. ROC curve analysis indicated that rounded 3.0 was the ideal CAR cutoff (area under the curve (AUC): 70.1%; sensitivity: 67.8%; specificity: 65.9%), which categorized the patients into CAR < 3.0 (*N* = 324) and CAR ≥ 3.0 (*N* = 327) groups. There were 308 (47.3%) and 343 (52.7%) patients without and with significant WL, respectively. The created CARWL groups were CARWL-0: CAR < 3.0 and WL ≤ 5.0%; CARWL-1: CAR < 3.0 and WL > 5.0%, or CAR ≥ 3.0 and WL ≤ 5.0%; and CARWL-2: CAR > 3.0 and WL > 5.0%. The Kaplan–Meier curves showed that the PFS (14.2 vs. 11.4 vs. 7.5 months; *P* < 0.001) and OS (37.3 vs. 23.6 vs. 12.8 months; *P* < 0.001) durations were gradually and significantly lowered from the CARWL-0 to CARWL-2 groups. The CARWL score's significant impacts on PFS and OS outcomes were found to be independent of the other variables in the multivariate analysis (*P* < 0.001, for each).

**Conclusions:**

Our findings indicate that the novel CARWL score, which accounts for pretreatment CAR and significant WL during the preceding 6 months, can reliably stratify newly diagnosed stage IIIC NSCLC patients into three groups with significantly different PFS and OS after definitive CCRT.

## 1. Introduction

Nearly one-third of all non-small-cell lung cancer (NSCLC) patients are diagnosed with stage III disease [1]. According to the 8^th^ edition of the American Joint Committee on Cancer (AJCC) staging system, stage IIIC NSCLC represents a patient group presenting with both locally (T_3-4_) and regionally (N_3_) advanced cancer (T_3-4_N_3_M_0_), namely, the group with the highest risk of distant metastases (DM) and the lowest survival results, excluding metastatic (M1) disease. Concurrent chemoradiotherapy (CCRT) is the current standard of treatment for these patients, based on multiple randomized clinical trials and meta-analysis results indicating that it outperforms sequential therapy [2–5]. Regardless, such patients' survival outcomes remain bleak even with aggressive CCRT, and other endeavors, such as escalating RT doses up to 74 Gy, have failed to enhance these outcomes [6]. Adjuvant immunotherapy is another recommended standard for these patients, but its usage remains challenging in many countries due to health insurance restrictions [7].

Although high local- and distant relapse rates due to relative resistance to currently available chemotherapy agents and RT appear to be the principal causes of poor results after radical CCRT, the clinical outcomes of such individuals vary dramatically even after identical CCRT regimens. The present TNM (tumor-node-metastasis) staging framework's sole focus on the original tumor's size and its local and regional expansion, without reference to tumor- and host-related biological variables, may explain such enormous discrepancies [8, 9]. Therefore, the introduction of new biological markers that may supplement the current TNM staging framework may aid in more advanced prognostic stratification and, presumably, tailored management of these patients.

The carcinogenesis process initiates systemic inflammation, the seventh hallmark of cancer, with a resultant increase in proinflammatory cytokines that stimulate the increased hepatic synthesis of C-reactive protein (CRP), a widely researched measure of inflammation [10–12]. Excessive inflammatory cytokine release not only increases capillary permeability and hence albumin loss, but it also reduces hepatocytes' ability to synthesize albumin [13]. As a result, higher CRP levels, and hence immunological activation, are associated with lower serum albumin levels: a clear sign of defective immunity, exacerbated inflammation, and malnutrition [14]. In this respect, increased CRP to albumin ratio (CAR) or its variant, Glasgow Prognostic Score (GPS), have previously been linked to a worse survival rate in NSCLC patients treated with various oncological strategies [15, 16].

Patients' performance status, quality of life (QOL), responsiveness to therapy, and prognosis have all been shown to be significantly impacted by weight loss (WL) at presentation, which is present to some degree in up to 80% of all NSCLC patients [17, 18]. The rates and intensity of treatment-related adverse symptoms such as early satiety, dysphagia, anorexia, nausea, vomiting, tiredness, esophagitis, diarrhea, and infections may also be increased by WL [19]. Such side effects may reduce the tolerance of CCRT and, as a result, the therapeutic response and prognosis. Significant WL, defined as WL > 5% in the last 6 months, is acknowledged as one of the main predictors of cancer-cachexia, which is unquestionably related to a poor prognosis in locally advanced NSCLC patients [20]. Furthermore, Evans et al. specified the components of CAR, namely, the levels of CRP (>5 mg/L) and albumin (<3.2 g/dL), among the biochemistry criteria for cancer-cachexia definition in the Washington consensus statement, bridging the gap between the WL and chronic inflammation [21].

Based on the encouraging basic and clinical evidence for CAR and WL in NSCLC patients, we hypothesized that the combination of baseline values of these two parameters could serve as a novel comprehensive biomarker in predicting the prognosis of stage IIIC NSCLC patients undergoing definitive pre-CCRT, by simultaneously providing the patient's immune, inflammation, and nutritional status. However, the prognostic value of combining the pretreatment CAR measurements and WL over the past 6 months, also known as the CARWL score, has not been studied in stage IIIC NSCLC patients who underwent CCRT, except for a conference abstract presented by our team [22]. Therefore, we planned to assess the prognostic significance of the novel CARWL score in IIIC NSCLC patients who received platinum-based definitive CCRT in the retrospective setting.

## 2. Patients and Methods

### 2.1. Patient Population

Patients in stage IIIC (AJCC 8^th^ ed.) who received CCRT with conventionally fractionated 60–66 Gy thoracic RT and at least one chemotherapy cycle concurrently between January 2010 and December 2020 were identified using an institutional retrospective database search. Patients had to fulfill the following criteria to be eligible for the study: aged between 18 and  80 years, Eastern Cooperative Oncology Group (ECOG) performance score of 0-1, body mass index (BMI > 20 kg/m^2^), pathological proof for NSCLC (adenocarcinoma (AC) or squamous-cell carcinoma (SCC)), stage IIIC disease by diagnostic computerized tomography (CT) and 18F-fluorodeoxyglucose positron emission tomography-CT (PET-CT), available pre-CCRT brain magnetic resonance imaging (MRI) scans, detailed chemotherapy and RT details, as well as complete blood count and biochemistry test results. Patients with established malignant pleural/pericardial effusion involved contralateral supraclavicular lymph nodes, a history of RT/chemotherapy, and inadequate pulmonary, cardiac, renal, or hepatic functions were considered ineligible for the study.

### 2.2. Ethics, Consent, and Permissions

Before any patient data were collected, the study design was approved by the Baskent University Medical Faculty's institutional review board under Project No. KA20/067. All patients provided written informed consent prior to the start of CCRT for the collection and analysis of blood samples and pathologic specimens as well as the publication of their results, either personally or through legally authorized representatives.

### 2.3. Treatment Details

We carried out all thoracic RT plans in line with our institutional care standards for newly diagnosed stage IIIC NSCLC patients, which dictate the use of coregistered diagnostic CT and PET-CT information. RT was delivered with megavoltage linear accelerators by utilizing the intensity-modulated RT (IMRT) technique. All target volume definitions, total and per fractional dosage specifications, normal tissue tolerance dose limits, and prescribed concomitant chemotherapies were the same as previously reported [23]. Elective nodal irradiation was not permitted as an institutional care standard for such patients. During the RT course, all patients received 60–66 Gy RT in 30–33 fractions (2 Gy per fraction) and 1–3 cycles of cisplatin/carboplatin plus one of docetaxel, paclitaxel, or vinorelbine combinations.

### 2.4. Assessment of C-Reactive Protein-to-Albumin Ratio and Weight Loss

The pretreatment CAR was calculated for each patient using CRP and albumin measures obtained on the first day of CCRT: CAR = CRP/Albumin. Similarly, the percentage difference between the weight measures acquired on the first day of CCRT and the weight stated by the patient for his/her measure 6 months before the start of CCRT was calculated. Significant WL was defined as WL > 5% in the previous 6 months, according to the Delphi criteria described by Fearon and colleagues [20].

### 2.5. Evaluation Treatment Response

Following completion of CCRT, scheduled follow-up visits and patient evaluations were performed every three months for the first two years and every six months or more frequently, if demanded, after that. For therapeutic response assessment, all patients were evaluated by complete blood count and biochemistry tests, PET-CT, or chest CT (following confirmation of metabolic complete response on PET-CT). The European Organization for Research and Treatment of Cancer (EORTC)-1999 guidelines were applied for this purpose. Additional radiologic and nuclear medicine imaging methods were exclusively employed in situations where there was a clinical suspicion of metastasis or for restaging of locoregionally recurrent NSCLC.

### 2.6. Statistical Methods

The primary endpoint of this research was to analyze if the CARWL score groups could achieve a statistically meaningful difference in overall survival (OS: time from the first day of CCRT to the date of death or the final visit). The secondary endpoint was progression-free survival (PFS: time from the first day of CCRT to the date of the first observation of disease progression or death or the final visit). Categorical variables were presented using percentage frequency distributions. Medians and ranges were used to express quantitative variables. We investigated the correlations between distinct groups using chi-square or Student's *t*-tests and Spearman correlations as indicated. Receiver operating characteristic (ROC) curve analysis was used to search for an optimal CAR cutoff that might divide the research population into two groups with substantially different OS and PFS findings. Patients were further divided into two groups based on whether they had significant WL before the CCRT, which was defined as WL > 5% in the last 6 months according to the Delphi criteria [19]. Kaplan–Meier estimates and log-rank tests were used to assess the potential impact of various risk variables on OS and PFS. The likely interactions between these variables and survival outcomes were investigated using the multivariate Cox proportional hazard model, with any two-sided *P* < 0.05 values being considered significant. For comparative subgroup analyses between three or more groups, the Bonferroni correction was utilized to reduce the chance-related false-positive results.

## 3. Results

We retrospectively assessed 651 patients with stage IIIC NSCLC (T_3-4_N_3_M_0_), 427 (65.6%) of whom were men. At presentation, the median age was 66 years (range: 27–79), and the majority of patients had ECOG 1 performance status (74.7%), AC histology (62.5%), T4 primary tumor stage (65.0%), and significant WL (52.7%), as depicted in Table 1.

At the end of the study, 234 patients (35.9%) were still alive and 108 (16.6%) showed no disease progression after a median follow-up of 20.3 months (range: 2.1–137.3 months). The median and 5-year PFS rates were 11.5 months [95% confidence interval (CI): 9.6–13.4 months] and 11.2%, respectively, whereas the matching OS rates were 23.1 months (95% CI: 19.4–26.8 months) and 18.2%. Among all 417 fatalities, widespread DM (*N* = 269; 64.5%) and uncontrolled locoregional primary (*N* = 107; 25.7%) were the leading causes of death, with the remaining 41 patients dying from comorbid conditions (*N* = 37; 8.9%) and late CCRT-related toxicities (*N* = 4; 0.9%).

**Figure 1:**
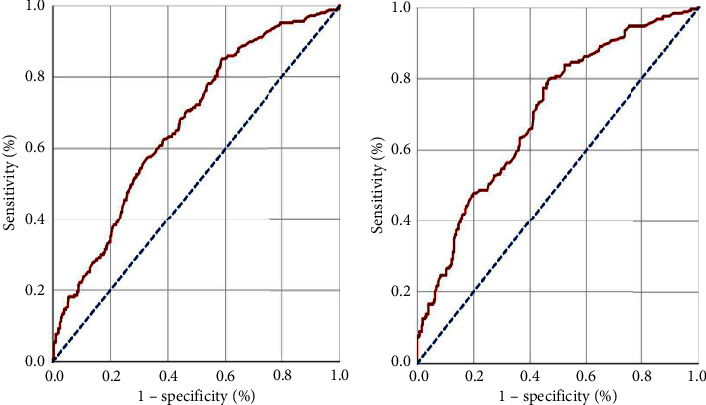
Results of the receiver operating characteristic curve analysis showing the association between the pretreatment CARWL score groups and survival outcomes: (a) progression-free survival; (b) overall survival.

Before proceeding to comparative analyses, ROC curve analysis was used to search the presence of ideal CAR cutoffs with that would interact with treatment results. Our search yielded significance at the cutoffs of 3.02 (area under the curve (AUC: 67.4%; sensitivity: 66.5%; specificity: 65.7%) and 2.96 (AUC: 70.1%; sensitivity: 67.8%; specificity: 65.9%) for PFS and OS, respectively (Figure 1). Because both cutoffs were remarkably close to 3.0, we decided to use this value as the common cutoff point for both PFS and OS comparisons. As a result, the research participants were split into two CAR groups: Group 1: CAR < 3.0 (*N* = 324) and Group 2: CAR ≥ 3.0 (*N* = 327). Comparisons between the two CAR groups revealed that the CAR ≥ 3.0 patients had significantly shorter median PFS (8.6 vs. 14.1 months; *P* < 0.001) and OS (16.7 vs. 31.0 months; *P* < 0.001) durations than their CAR < 3.0 counterparts (Table 2). Indicating that the outcomes were steadily worsening with a high CAR value over time, the actuarial 5-year and 8-year PFS and OS rates were likewise inferior in the CAR ≥ 3.0 patients' group (Table 2).

In keeping with the study's objectives, we further categorized the entire research population into two WL groups based on Fearon's criteria [24]: WL-1: Significant WL absent (*N* = 308; 47.3%) and WL-2: Significant WL (>5%) present (*N* = 343; 52.7%). Comparative survival analysis results showed that the patients presenting with significant WL had significantly shorter median PFS (9.4 vs. 13.2 months; *P* < 0.001) and OS (17.1 vs. 30.7 months; *P* < 0.001) durations than those without significant WL (Table 2). Likewise, patients with significant WL had numerically inferior PFS and OS rates at 5 and 8 years (Table 2).

**Figure 2:**
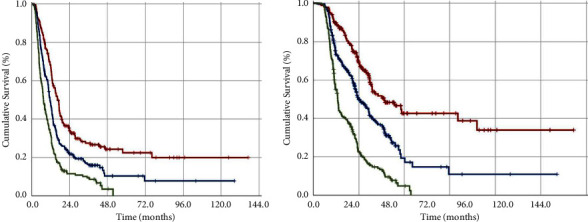
Survival outcomes based on three pretreatment CARWL score groups: (red line: CARWL-0: CAR < 3.0 and WL ≤ 5.0%; dark blue line: CARWL-1: CAR < 3.0 and WL > 5.0%, or CAR ≥ 3.0 and WL ≤ 5.0%; and dark green line: CARWL-2: CAR > 3.0 and WL > 5.0%): (a) progression-free survival; (b) overall survival.

Results of the univariate analysis indicated that, except for the pretreatment CAR and WL groups, none of the other factors had a statistically significant impact on the PFS or OS outcomes of the study cohorts (Table 3). As a result, we divided patients into four groups based on their pretreatment CAR and WL: Group-1: CAR < 3.0 and WL ≤ 5.0%, Group-2: CAR < 3.0 and WL > 5.0%, Group-3: CAR ≥ 3.0 and WL ≤ 5.0%, and Group-4: CAR > 3.0 and WL > 5.0%, respectively. Nevertheless, because the PFS and OS outcomes of Groups 2 and 3 were statistically insignificant, we merged them into one group and created the three-tiered CARWL score: CARWL-0: CAR < 3.0 and WL ≤ 5.0%; CARWL-1: CAR < 3.0 and WL > 5.0%, or CAR ≥ 3.0 and WL ≤ 5.0%; and CARWL-2: CAR > 3.0 and WL>5.0% (Table 4). As shown in Figure 2 and Table 2, results of Kaplan–Meier estimates showed that the PFS (14.2 vs. 11.4 vs. 7.5 months; *P* < 0.001) and OS (37.3 vs. 23.6 vs. 12.8 months; *P* < 0.001) outcomes were gradually and significantly lowering from CARWL-0 to CARWL-2 score groups, despite nearly equal distributions of baseline disease and patient characteristics between the groups. The results of the multivariate analysis indicated that the CARWL score's significant impacts on PFS and OS outcomes were independent of the other variables (Table 3), establishing the novel CARWL score as a robust immune-inflammation and nutritional score for stage IIIC NSCLC patients. Further investigation into the source of this disparity found that the earlier and much larger rates of DM in the higher CARWL score groups were the most likely explanation since locoregional control rate differences were not statistically significant. Affirming this explanation, DM was detected in 157 (76.2%) of 206 CARWL-2 patients, with 116 (73.9%) of them manifesting in the first 9 months of CCRT, whereas corresponding rates for CARWL-1 and CARWL-0 patients were 40.6% and 29.2%, respectively (*P* < 0.001).

## 4. Discussion

Stage IIIC NSCLC (T_3-4_N_3_M_0_ disease) is the penultimate stage before oligometastatic (IVA) or extensively metastatic (IVB) cancer in the current TNM staging framework (AJCC 8^th^ ed). Survival outcomes following almost similar therapies, on the other hand, may differ dramatically between individuals, which might be connected to biological differences such as systemic immune and inflammation status, as well as nutritional condition. In this respect, the present study represents a first by investigating the prognostic usefulness of the combination of pre-CCRT CAR and significant WL (>5%) over the previous 6 months, namely, the CARWL score, in patients who underwent definitive CCRT for stage IIIC NSCLC. The two main findings of this study were as follows: first, the pre-CCRT CARWL score was able to stratify stage IIIC patients into three groups with substantially different PFS (*P* < 0.001) and OS (*P* < 0.001), confirming its prognostic usefulness. Second, whilst CARWL-1 patients had essentially identical outcomes to typical IIIC patients, PFS and OS results for CARWL-0 and CARWL-2 patients were comparable to stage II and IVB NSCLC patients, implying the need for adjustment of the staging and individual treatment algorithms.

Systemic inflammation, the seventh hallmark of cancer, is triggered by carcinogenic processes, with a commensurate surge in proinflammatory cytokine synthesis and release [24]. As a result of this triggering action, cytokines like tumor necrosis factor-*α* (TNF-*α*) and interleukin-6 (IL-6) enhance hepatic CRP production, leading to increased systemic inflammation and decreased albumin synthesis [12, 24]. Additionally, cytokine-induced increased capillary permeability further lowers the albumin levels due to leakage into the interstitial space [13]. Elevated CRP and accompanying exacerbated inflammatory responses are, thus, associated with lower serum albumin levels [25]. CRP and albumin are not just the components of the immune-inflammation index CAR, but they are also strong predictors of liver function, malnutrition, and eventual WL in cancer patients, which can lead to deadly cachexia [13]. CAR or its GPS variant has already been related to worse survival outcomes in NSCLC patients [15, 16]. Similarly, the Washington consensus statement identified CAR components CRP (>5 mg/L) and albumin (<3.2 g/dL) as biochemical indicators of cancer-cachexia [21]. When these facts are combined with the principal determinant of cancer-cachexia definition, namely, the significant WL in the previous 6 months, it appears that there is a strong link between CAR components, WL, persistent inflammation, defective immunity, cancer-cachexia, and cancer progression, all of which result in poor treatment response and patient prognosis. Confirming this connection, we found that 343 (52.7%) and 327 (50.2%) patients had significant WL and high CAR levels, respectively. Furthermore, 206 (31.6%) of those patients had both high CAR and significant WL simultaneously, forming the current study's CARWL-2 cohort. Hence, in the lack of similarly designed NSCLC research, our study's results seem to establish the link between persistent inflammation and resultant WL (likely the most critical phase in the cancer-cachexia cascade) and poor clinical outcomes.

Although we revealed that a high CAR value and significant WL before CCRT are two notable predictors of survival outcomes in stage IIIC patients, the most noteworthy finding of our research was the demonstration of the novel CARWL score's unique capability to stratify these patients into three separate PFS and OS groups. Because the outcomes of the CARWL-0, CARWL-1, and CARWL-2 score groups were very similar to those reported for stage II, IIIC, and IVB patients, our findings appear to substantiate the novel CARWL score as a reliable and potent prognostic biomarker for such patients, given the inability of the traditional TNM staging framework for such discrimination across patients in the same disease stage. Furthermore, in the absence of 5-year survivors, a median OS of only 12.8 months in CARWL-2 patients could clinically indicate inflammation-induced chemoresistance and/or radioresistance of the primary tumor and occult systemic metastases that are beyond the detection capability of current staging tools, including the FDG-PET-CT [26, 27], emphasizing the urgent need to revise standard treatment protocols for these patients. Confirming the presence of resistant occult metastases before the onset of CCRT, DM was detected in 157 (76.2%) of 206 CARWL-2 patients, with 116 (73.9%) of them manifesting in the first 9 months of CCRT. This discovery may clinically imply the implementation of more sophisticated staging tools, such as liquid biopsy, which may aid in the more accurate staging of these patients who require more intensive systemic therapies while sparing the already metastatic patients from the unavailing toxicities of radical CCRT. Alternatively, our findings could indicate that adding highly effective anti-inflammatory agents, such as the cyclooxygenase-2 inhibitor celecoxib, to the treatment algorithm of CARWL-2 patients may improve their results, given Komaki and colleagues' impressive results in unresectable locally advanced NSCLC patients [28].

Our research has certain limitations. First, because it was a single-center retrospective cohort analysis, the findings presented here are prone to negative consequences of sampling bias. Second, these findings may not apply to other stage IIIC NSCLC patients, such as those with an ECOG 2 performance score and/or receiving different RT or chemotherapy regimens, since we included only patients with an ECOG performance score of 0-1 who underwent indistinguishable staging and treatment processes. Third, we did not run CARWL group-specific correlations with other immune-inflammation and cachexia indicators such as blood-borne platelets, neutrophils, monocytes, lymphocytes, and chemokines like interleukin-6, which could have disclosed the precise mechanisms underlying the novel CARWL score and the survival outcomes of stage IIIC NSCLC patients. And fourth, current discoveries should be interpreted with caution because, albeit statistically insignificant, discrepancies in salvage therapies may have inadvertently benefited one CARWL group over the others. As a result, until the results of well-conducted large-scale research are available, our findings should be viewed with caution and regarded as hypothesis generators rather than firm recommendations. Yet, when viewed through Clark's prognostic factor definition [29], our findings appear to mark the CRAWL score as a reputable, simple to achieve and calculate, factually quantifiable, reproducible, and reasonably priced novel prognostic score for stage IIIC NSCLC patients undergoing radical CCRT.

## 5. Conclusions

Although confirmatory research is crucial to back up present discoveries, the results of this hypothesis-generating research revealed that the novel CARWL score, which integrates pretreatment CAR and significant WL over the previous 6 months, can reliably stratify newly diagnosed stage IIIC NSCLC patients into three groups with significantly different PFS and OS after definitive CCRT.

## Figures and Tables

**Table 1:** Baseline patient and disease characteristics.

Covariate	All patients (*N* = 651)	CARWL-0 (*N* = 186)	CARWL-1 (*N* = 259)	CARWL-2 (*N* = 206)	*P* value
Median age, y (range)	66 (27–79)	65 (29–79)	67 (33–79)	64 (31–79)	0.78
Age group, y (%)					
≤70 years	466 (71.6)	131(70.4)	188 (72.6)	147 (71.4)	0.81
>70 years	185 (28.4)	55 (29.6)	71 (27.4)	59 (28.6)	
Gender, *n* (%)					
Female	224 (34.4)	61 (32.8)	88 (34.0)	75 (36.4)	0.57
Male	427 (65.6)	125 (67.2)	171 (66.0)	131 (63.6)	
ECOG, *n* (%)					
0	165 (25.3)	49 (26.3)	77 (29.7)	39 (18.9)	0.24
1	486 (74.7)	137 (73.7)	182 (70.3)	167 (81.1)	
Histology, *n* (%)					
SCC	244 (37.5)	69 (37.1)	105 (40.5)	70 (34.0)	0.27
AC	407 (62.5)	117 (62.9)	154 (59.5)	136 (66.0)	
T-stage, *n* (%)					
3	228 (35.0)	67 (36.0)	90 (34.7)	71 (34.5)	0.53
4	423 (65.0)	119 (64.0)	169 (65.3)	135 (65.5)	

CARWL: C-reactive protein-to albumin ratio and weight loss; ECOG: Eastern Cooperative Oncology Group; SCC: squamous-cell carcinoma; AC: adenocarcinoma; T-stage: tumor stage.

**Table 2:** Survival outcomes per C-reactive protein-to albumin ratio (CAR), weight loss (WL), and combined C-reactive protein-to albumin ratio and weight loss (CARWL) score subgroups.

Outcome	All patients (*N* = 651)	CAR < 3.0 (*N* = 324)	CAR ≥ 3.0 (*N* = 327)	*P* value	WL ≤ 5% (*N* = 308)	WL > 5% (*N* = 343)	*P* value	CARWL-0 (*N* = 186)	CARWL-1 (*N* = 259)	CARWL-2 (*N* = 206)	*P* value^*∗*^
PFS											
Median (mo.)	11.5	14.1	8.6	<0.001	13.2	9.4	<0.001	14.2	11.4	7.5	<0.001
5-year (%)	11.2	17.6	4.6		16.4	4.5		22.5	10.3	0	
8-year (%)	9.7	14.6	4.6		16.4	2.2		20.0	7.7	0	
OS											
Median (mo.)	23.1	31.0	16.7	<0.001	30.7	17.1	<0.001	37.3	23.6	12.8	<0.001
5-year (%)	18.2	32.4	4.7		31.8	5.8		42.6	14.7	0	
8-year (%)	14.3	24.5	4.7		26.1	2.9		33.9	11.0	0	

^*∗*^Bonferoni corrected *P* value <0.017 for significance. *Note*. CARWL-0: CAR < 3.0 and WL ≤ 5.0%; CARWL-1: CAR < 3.0 and WL > 5.0%, or CAR ≥ 3.0 and WL ≤ 5.0%; and CARWL-2: CAR > 3.0 and WL > 5.0%. PFS: progression-free survival; OS: overall survival; mo.: months.

**Table 3:** Univariate and multivariate analysis outcomes.

Characteristics	Patients (*N*)	Median PFS (moths)	Univariate *P* value	Multivariate *P* value	Median OS (months)	Univariate *P* value	Multivariate *P* value
Age group							
≤70 years	466	11.8	0.47	—	24.1	0.38	—
>70 years	185	10.7			22.6		
Gender							
Female	224	10.9	0.49	—	22.3	0.39	—
Male	427	11.8			23.9		
ECOG							
0	486	11.8	0.74	—	24.1	0.63	—
1	165	11.0			22.7		
Histology							
SCC	244	11.2	0.59	—	22.0	0.68	—
AC	407	12.1			23.9		
T-stage							
3	228	12.7	0.19	—	24.6	0.16	—
4	423	10.9			22.2		
RT dose							
60 Gy	303	11.7	0.89	—	23.4	0.76	—
66 Gy	348	11.4			22.9		
Significant WL							
Absent	308	13.2	<0.001	<0.001	30.7	<0.001	<0.001
Present	343	9.4			17.1		
CAR group							
<3.0	324	14.1	<0.001	<0.001	31.0	<0.001	<0.001
≥3.0	327	8.6			16.7		
CARWL group							
0	186	14.2	<0.001	<0.001	37.3	<0.001	<0.001^*∗*^
1	259	11.4			23.6		
2	206	7.5			12.8		

^*∗*^Bonferoni corrected *P* value <0.017 for significance. Groups 1 vs. 2: *P* < 0.001. Groups 1 vs. 3: *P* < 0.001. Groups 2 vs. 3: *P* < 0.001. *Note*. CARWL-0: CAR < 3.0 and WL ≤ 5.0%; CARWL-1: CAR < 3.0 and WL > 5.0%, or CAR ≥ 3.0 and WL ≤ 5.0%; and CARWL-2: CAR > 3.0 and WL > 5.0%. PFS: progression-free survival; OS: overall survival; ECOG: Eastern Cooperative Oncology Group; SCC: squamous-cell carcinoma; AC: adenocarcinoma; T-stage: RT: radiotherapy; Gy: gray; tumor stage; CAR: C-reactive protein-to albumin ratio; WL: weight loss; CARWL: C-reactive protein-to albumin ratio and weight loss score.

**Table 4:** Definition of CARWL (C-reactive protein-to albumin ratio (CAR) and weight loss (WL)) score groups.

Group	Definition
CARWL-0	CAR < 3.0 and WL ≤ 5.0%
CARWL-1	CAR < 3.0 and WL > 5.0% or CAR ≥ 3.0 and WL ≤ 5.0%
CARWL-2	CAR > 3.0 and WL > 5.0%

## Data Availability

For researchers who satisfy the criteria for access to sensitive data, the datasets utilized and/or analyzed during the current study are accessible from the Baskent University Department of Radiation Oncology Institutional Data Access: adanabaskent@baskent.edu.tr.
